# A Functionally Defined *In Vivo* Astrocyte Population Identified by c-Fos Activation in a Mouse Model of Multiple Sclerosis Modulated by S1P Signaling: Immediate-Early Astrocytes (*ieAstrocytes*)

**DOI:** 10.1523/ENEURO.0239-18.2018

**Published:** 2018-09-24

**Authors:** Aran Groves, Yasuyuki Kihara, Deepa Jonnalagadda, Richard Rivera, Grace Kennedy, Mark Mayford, Jerold Chun

**Affiliations:** 1 Sanford Burnham Prebys Medical Discovery Institute, La Jolla, CA 92037; 2 Department of Psychiatry, University of California San Diego, La Jolla, CA 92037

**Keywords:** Astrocyte, c-Fos GPCR, lipid mediator, neuroinflammation, S1P

## Abstract

Astrocytes have prominent roles in central nervous system (CNS) function and disease, with subpopulations defined primarily by morphologies and molecular markers often determined in cell culture. Here, we identify an *in vivo* astrocyte subpopulation termed immediate-early astrocytes (*ieAstrocytes*) that is defined by functional c-Fos activation during CNS disease development. An unbiased screen for CNS cells showing c-Fos activation during experimental autoimmune encephalomyelitis (EAE), a mouse model for multiple sclerosis (MS), was developed by using inducible, TetTag c-Fos reporter mice that label activated cells with a temporally stable, nuclear green fluorescent protein (GFP). Four-dimensional (3D over time) c-Fos activation maps in the spinal cord were produced by combining tissue clearing (iDISCO) and confocal microscopy that identified onset and expansion of GFP^+^ cell populations during EAE. More than 95% of the GFP^+^ cells showed glial fibrillary acidic protein (GFAP) immunoreactivity—in contrast to absent or rare labeling of neurons, microglia, and infiltrating immune cells—which constituted *ieAstrocytes* that linearly increased in number with progression of EAE. *ieAstrocyte* formation was reduced by either astrocyte-specific genetic removal of sphingosine 1-phosphate receptor 1 (S1P_1_) or pharmacological inhibition by fingolimod (FTY720), an FDA-approved MS medicine that can functionally antagonize S1P_1_. *ieAstrocyte*s thus represent a functionally defined subset of disease-linked astrocytes that are the first and predominant CNS cell population activated during EAE, and that track with disease severity *in vivo*. Their reduction by a disease-modifying agent supports their therapeutic relevance to MS and potentially other neuroinflammatory and neurodegenerative diseases.

## Visual Abstract

**Figure:**
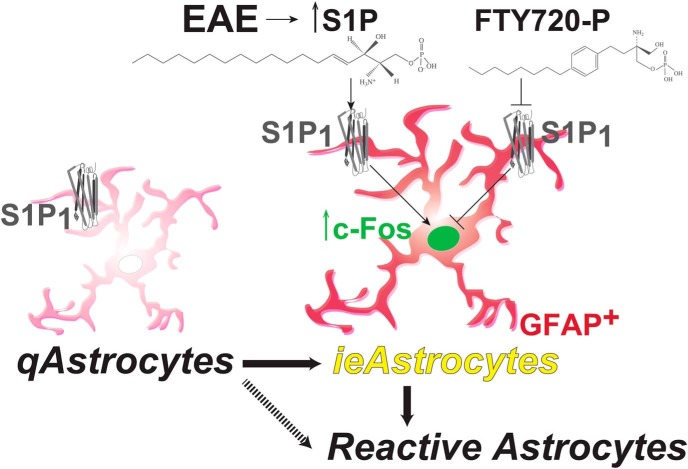

## Significance Statement

A new, functionally defined *in vivo* subpopulation of astrocytes termed immediate-early astrocytes (*ieAstrocyte*s) was identified as the first and predominant CNS cell type showing c-Fos activation, in an animal model of multiple sclerosis (MS). *ieAstrocyte*s track with disease severity. The approved MS drug fingolimod (FTY720, an S1P receptor modulator) reduced *ieAstrocyte* formation as well as clinical disease. Agents that prevent or reduce *ieAstrocytes* could represent a new strategic target for treating MS and other neuroinflammatory and neurodegenerative brain disorders.

## Introduction

Neuroinflammation in the central nervous system (CNS), as epitomized by multiple sclerosis (MS), involves activation of resident immune-competent cells, including astrocytes (Cekanaviciute and Buckwalter, 2016), along with infiltration of peripheral immune cells (González and Pacheco, 2014), which have been associated with multiple CNS pathologies such as MS (Noseworthy et al., 2000), Alzheimer’s disease (Acosta et al., 2017), and neuropsychiatric disorders (Trepanier et al., 2016). MS is a prototypical neuroinflammatory disease that also includes demyelination and neurodegeneration in the CNS (Noseworthy et al., 2000). Its cause is unclear; however, in animal models such as experimental autoimmune encephalomyelitis (EAE), immune cells, particularly CD4^+^ T cells, infiltrate the CNS to initiate demyelination and neurodegeneration. Both EAE and human MS can be therapeutically treated by the FDA-approved medicine fingolimod (FTY720; Herr and Chun, 2007; Chun and Hartung, 2010; Lee et al., 2010; Chun and Brinkmann, 2011; Noguchi and Chun, 2011).

FTY720 is a chemical analog of a naturally occurring lipid, sphingosine. It can be phosphorylated by endogenous sphingosine kinases to produce FTY720-phosphate (FTY720-P), an analog of the bioactive lysophospholipid known as sphingosine 1-phosphate (S1P). S1P signals through five receptor subtypes (S1P_1–5_), all of which are G protein-coupled receptors (GPCRs; Kihara et al., 2014) that can have roles in neuroinflammation (Gardell et al., 2006; Herr and Chun, 2007; Moon et al., 2015; Proia and Hla, 2015; Tsai and Han, 2016). FTY720-P also signals through S1P receptors, engaging four of the five known subtypes (S1P_1_, S1P_3_, S1P_4_, and S1P_5_; Kihara et al., 2015). In particular, S1P_1_ is thought to be mechanistically important in MS in that a functional antagonism of S1P_1_ reduces lymphocyte egress from secondary lymphoid organs, resulting in a decreased number of pathogenic lymphocytes entering the CNS (Mandala et al., 2002; Arnon et al., 2011). Complementing these immune effects, a direct CNS mechanism of action (MOA) for fingolimod has been implicated (Choi et al., 2011), which also involves functional antagonism of S1P_1_ expressed by astrocytes, based on similar results observed following the genetic removal of S1P_1_ from astrocytes, which both resulted in reduced EAE severity and elimination of FTY720 efficacy, despite intact lymphocyte trafficking effects (Choi et al., 2011). S1P signaling is also implicated in controlling astrocyte functions that include gap junction formation (Rouach et al., 2006), migration (Mullershausen et al., 2007), cytokine production (Dusaban et al., 2017), and nitric oxide production (Colombo et al., 2014) via S1P_1_ and/or S1P_3_, all of which support S1P-mediated roles for astrocytes in EAE and MS.

To identify CNS cell types altered during neuroinflammatory processes, we developed an unbiased *in vivo* screen based on transcription of c-Fos (Bullitt, 1990), an immediate-early gene (IEG) that is rapidly transcribed in response to cellular stimuli, independent of *de novo* protein synthesis (Lau and Nathans, 1987). c-Fos can be activated in astrocytes (McNaughton and Hunt, 1992; Anderson et al., 1994; Morishita et al., 2011; Yester et al., 2015), microglia (Eun et al., 2004), and oligodendrocytes (Muir and Compston, 1996), as well as within neurons associated with memory (Matsuo et al., 2008). We adapted a conditional c-Fos reporter mouse that historically marks cells with nuclear GFP, which could be followed in four-dimensional (3D over time) analyses. Remarkably, more than 95% of disease-activated cells *in vivo* were astrocytes that we named immediate-early astrocytes (*ieAstrocytes*). Here, we describe the *in vivo* screen used to identify the *ieAstrocytes* produced by EAE challenge, including their temporal and spatial patterns and their quantitative modification by S1P receptor signaling, demonstrated through the use of genetic and pharmacological interventions.

## Materials and Methods

### Mice

All animal protocols were approved by the Sanford Burnham Prebys Medical Discovery Institute IACUC and conformed to National Institutes of Health guidelines and public law. The TetTag-cFos mice were generated by crossing Tg(Fos-tTA)1Mmay (MGI:5014071) and Tg(tetO-HIST1H2BJ/GFP)47Efu/J (RRID:IMSR_JAX:005104) mice, which expressed a GFP-histone H2B fusion protein (GFP-H2B) under a tetO promoter controlled by a c-Fos inducible tetracycline transactivator (tTA) protein as described earlier (Tayler et al., 2011). S1P_1_^flox/flox^:hGFAP-cre mice (Choi et al., 2011) were crossed with TetTag mice for multiple generations to generate astrocyte-specific S1P_1_ deletion (S1P_1_-AsCKO^fos^, TetTag:S1P_1_^flox/flox^:hGFAP-cre) and their littermate controls (WT^fos^, TetTag:S1P_1_^flox/flox^). TetTag-cFos mice were maintained on doxycycline diet (DOX, 40 mg/kg; Bio-Serv) throughout breeding, birth, and development to prevent GFP-H2B expression until experimental examination.

### EAE

EAE was induced in 7- to 13-wk-old female mice (Kihara et al., 2009). Briefly, mice were immunized subcutaneously with 150 µg myelin oligodendrocyte glycoprotein 35–55 (MOG_35–55_) (MEVGWYRSPFSRVVHLYRNGK, EZBiolab) in PBS and complete Freund’s adjuvant containing 4 mg/mL M. Tuberculosis H37Ra (Difco) with or without intraperitoneal injection of 250 ng pertussis toxin (List Biological Laboratories) on days 0 and 2. Daily clinical scores corresponding to the most severe sign observed were given as follows: 0, no sign; 0.5, mild loss of tail tone; 1.0, complete loss of tail tone; 1.5, mildly impaired righting reflex; 2.0, abnormal gait and/or impaired righting reflex; 2.5, hindlimb paresis; 3.0, hindlimb paralysis; 3.5, hindlimb paralysis with hind body paresis; 4.0, hind- and forelimb paralysis; and 4.5, death or severity necessitating euthanasia. FTY720 was administered via gavage (1 mg/kg; Novartis).

### Histologic analyses (cryosectioning)

Mice were euthanized with an overdose of isoflurane followed by rapid dissection of spinal cords (SCs). SCs were cut in three sections, embedded in Tissue-Tek Optimal Cutting Temperature (OCT; Ted Pella) compound, and frozen on crushed dry ice. Cryostat sections (16 μm) were collected on Superfrost Plus microscope slides (Thermo Fisher Scientific) and fixed (10 min) with freshly prepared 4% paraformaldehyde (PFA; Sigma) in PBS. Sections were washed three times in TBS with 0.3 M glycine, permeabilized with 0.1% Triton X-100 in TBS, blocked with species-appropriate serum, and immunolabeled with chicken anti-GFAP (1:1000 dilution, Neuromics, Cat #CH22102, RRID:AB_10014322), rabbit anti-NeuN (1:400 dilution, Millipore, Cat #MABN140, Clone #27-4, RRID:AB_2571567), mouse anti-Olig1,2,3 (1:100 dilution, Neuromics, Cat #MO15059, Clone #257224, RRID:AB_1620409), rabbit anti-Iba1 (1:500 dilution, Wako, Cat #019-19741, Clone #NCNP24, RRID:AB_839504), and hamster anti-CD3e (1:500 dilution, BD Biosciences, Cat# 553057, Clone #145-2C11, RRID:AB_394590). Antigen retrieval with Diva Decloaker (Biocare) as per manufacturer’s instructions was performed in place of permeabilization for Olig1,2,3 staining. Sections were washed in PBS, labeled with secondary antibodies conjugated with Alexa Fluor 568 (1:2000 dilution, Thermo Fisher Scientific; Cat# A-11041, RRID:AB_2534098; Cat# A-11031, RRID:AB_144696; Cat# A-11036, RRID:AB_10563566) or DyLight 649 (1:2000 dilution, BioLegend, Cat# 405505, RRID:AB_1575122), counterstained with DAPI (1:10,000 dilution, Thermo Fisher Scientific, Cat# D1306, RRID:AB_2629482), and coverslipped with Vectashield Antifade Mounting Medium (Vector). Sections were visualized and images acquired on a Zeiss Imager 1D microscope (Axiovision 4.8, RRID:SCR_002677), a Zeiss ApoTome.2 (Zen 2 Blue Edition, RRID:SCR_013672), or a Nikon A1^+^ (NIS-Elements v4.4, RRID:SCR_014329).

### Histologic analyses (iDISCO)

Immunolabeling-enabled three-dimensional imaging of solvent-cleared organs (iDISCO) was employed to visualize GFP-H2B in intact SCs (Renier et al., 2014). Mice were euthanized with an overdose of isoflurane anesthesia, then fixed by intracardiac perfusion of PBS followed by 4% PFA in PBS. SCs were dissected out and postfixed overnight in 4% PFA in PBS; washed in PBS, 50% methanol in PBS, 80% methanol in PBS, and 100% methanol; and bleached in 20% DMSO in methanol containing 5% H_2_O_2_ at 4°C. Samples were washed again in 100% methanol, 20% DMSO in methanol, 80% methanol in PBS, 50% methanol in PBS, PBS, and PBS containing 0.2% Triton X-100 and incubated at 37°C in PBS containing 0.2% Triton X-100/20%DMSO/0.3 M glycine. Samples were blocked in PBS containing 0.2% Triton X-100/10% DMSO/6% goat serum at 37°C, washed in PBS containing 0.2% Tween 20 and 10 µg/ml heparin (PTwH), and incubated in a rabbit anti-GFP antibody (1:1000 dilution, MBL International Cat# 598, RRID:AB_591819) in PTwH at 37°C. Samples were washed in PTwH, then incubated in anti-rabbit Alexa Fluor 488 (1:1000 dilution, Thermo Fisher Scientific Cat# A-11008, RRID:AB_143165). Samples were washed again in PTwH before tissue clearing. SC samples were incubated in 50% v/v tetrahydrofuran/H_2_O (THF, Sigma), 70% THF in H_2_O, 80% THF in H_2_O, 100% THF, dichloromethane (Sigma), and finally dibenzyl ether (DBE). Cleared SCs were placed in a microscope chamber made with two to three stacked Fastwells (Research Products International), filled with DBE, and coverslipped. The intact SCs were imaged on a Nikon A1^+^ confocal microscope using *z*-steps of 10 µm under 10× objective. NIS-Elements (v4.3, RRID:SCR_014329) was used to three-dimensionally reconstruct the entire SC. Obtained images were analyzed with ImageJ (NIH, RRID:SCR_003070) to count the number of signals, areas, and average area of signals.

### Flow cytometry

SCs were rapidly dissected and frozen in liquid nitrogen. Samples were equilibrated to 4°C and dounce-homogenized in a nucleus extraction buffer made with 0.32 M sucrose/5 mM CaCl_2_/3 mM Mg(CH_3_COO)_2_/0.1 mM EDTA/20 mM Tris-HCl, pH 8.0/0.1% Triton X-100 in DEPC-treated H_2_O. Homogenized samples were filtered (50 μm; Celltrics, Sysmex) and washed in DEPC-treated PBS containing 2 mM EDTA (PBSE-d). Nuclei were purified by centrifugation at 3250 × *g* for 12 min in an isosmotic iodixanol gradient made of a 35%, 10%, and 5% OptiPrep (Sigma) in DEPC-treated H_2_O containing 20 mM tricine-KOH (pH 7.8), 25 mM KCl, and 30 mM MgCl_2_ (Graham, 2001). Nuclei were recovered in the 35%-10% interface, washed in PBSE-d, and immunolabeled with rabbit anti-NeuN antibody (1:400 dilution, Millipore, Cat #MABN140, Clone #27-4, RRID:AB_2571567) in 1% bovine serum albumin (BSA)/PBSE-d for 20 min. Samples were washed in PBSE-d and labeled with a goat anti-rabbit APC-conjugated secondary antibody (1:500 dilution, Thermo Fisher Scientific, Cat# A10931, RRID:AB_10562534) and DAPI (1:5000 dilution, Thermo Fisher Scientific, Cat# D1306, RRID:AB_2629482) in 1%BSA/PBSE-d for 10 min. Samples were washed in PBSE-d and suspended in PBSE-d. Nuclear populations were analyzed and sorted on a BD FACSAria II. Gating was performed as follows: (1) DAPI positive, (2) size and granularity consistent with nuclei by forward-scatter area (FSC-A) and side-scatter area (SSC-A), and (3) single nuclei selected by both FSC-A and forward-scatter height (FSC-H) and SSC-A and side-scatter height (SSC-H). Analysis was performed on FlowJo (10.0.8r1, RRID:SCR_008520).

### Statistical analysis

Results are expressed as means ± SEM. Data were analyzed statistically by means of ANOVA with indicated *post hoc* tests as appropriate, using GraphPad PRISM software (RRID:SCR_002798). Values of *p* < 0.05 were considered to be statistically significant.

## Results

### A new, *in vivo* c-Fos screen detects CNS cells activated by neuroinflammation

**Figure 1.**
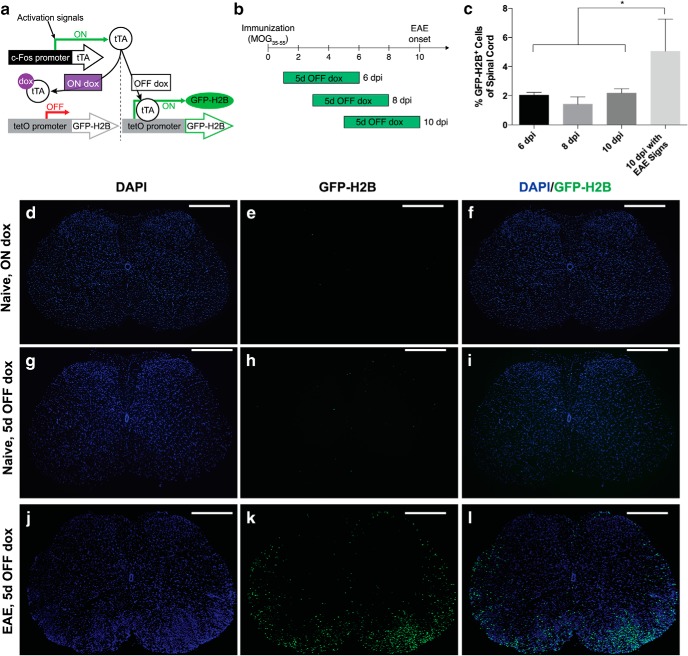
c-Fos reporter mice display increased c-Fos activation in EAE spinal cords (SCs). ***a***, Schematic representation of doxycycline (dox)-controlled GFP-H2B expression. Dox removal (off dox) allows tTA (tetracycline-transactivator) expression under a c-Fos promoter to drive fused green fluorescent protein-human histone H2B (GFP-H2B) expression. ***b***, Experimental scheme of 5-d dox removal period. Average onset of EAE in WT^fos^ controls was 10.6 ± 1.07 days postimmunization (dpi; *n* = 7). ***c***, GFP-H2B^+^ population in the spinal cord was determined by FCM (*n* = 4 animals, mean ± SEM, *p < 0.05, one-way ANOVA with Bonferroni’s *post hoc* test). ***d–l***, Representative spinal cord coronal sections. In the naive SC, DOX inhibited extra-experimental tagging of GFP^+^ cells. Virtually no GFP-H2B was observed (***d–f***). On DOX removal, there were sparse and dispersed GFP^+^ cells in the naive SC over 5 d (***g****–****i***). EAE induced robust GFP signals in the SC at 5 days post-onset (dpo) that were clustered in lesions and along the periphery (**j**–**l**). Scale bar, 500 μm.

**Figure 2.**
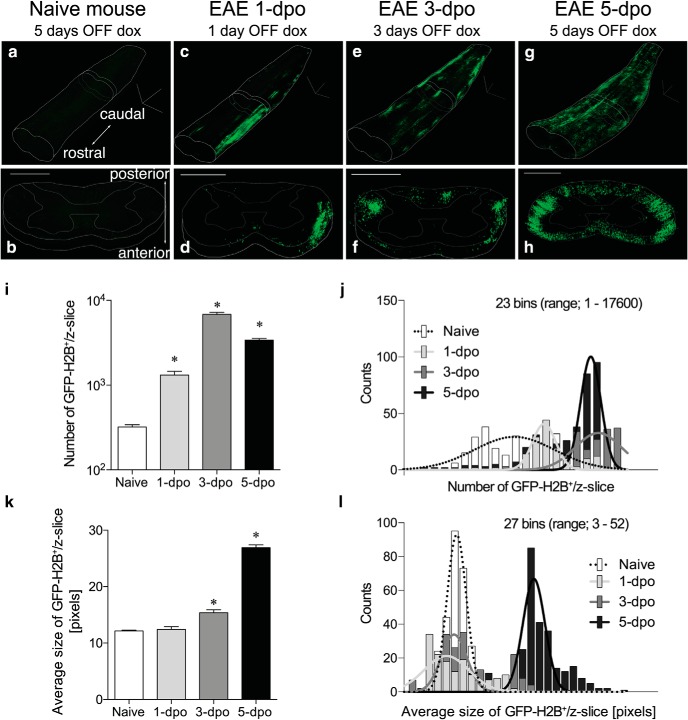
c-Fos activation is temporally and spatially propagated in EAE SC. ***a****–****h***, Representative 3D imaging of lower (lumbar region) SCs in naive (***a*** and ***b***) and EAE-induced reporter mice of 1 (***c*** and ***d***), 3 (***e*** and ***f***), and 5 (***g*** and ***h***) days post-onset (dpo, *n* = 2–5 animals for each dpo). ***a***, ***c***, ***e***, ***g***, 3D-reconstructed images. ***b***, ***d***, ***f***, ***h***, Coronal sections. Scale bar, 500 μm. ***i***, The number of GFP-H2B^+^ nuclei in the SCs (mean ± SEM, *p < 0.001 versus naive, Kruskal–Wallis test with Dunn’s *post hoc* test, data were compiled from 273, 149, 185, and 381 *z*-slices from 3, 2, 2, and 4 animals, respectively). ***j***, Distribution of the number of GFP-H2B^+^ nuclei per *z*-slice as shown in ***i***. Lines represent Gaussian fits to the data. ***k***, Average size of GFP-H2B^+^ signals (mean ± SEM, *p < 0.001 versus naive, Kruskal–Wallis test with Dunn’s *post hoc* test, data were compiled from 273, 149, 185, and 381 *z*-slices from 3, 2, 2, and 4 animals, respectively). ***l***, Distribution of the average size of GFP-H2B^+^ signals per *z*-slice shown in ***k***. Lines represent Gaussian fits to the data.

We hypothesized that EAE would induce c-Fos expression in key, disease-relevant cells. An unbiased screen was developed using the c-Fos-inducible and doxycycline-regulated TetTag cFos system that labels cell nuclei with GFP permanently (Fig. 1*a*). This system was used previously to identify neurons involved in learning and memory (Matsuo et al., 2008). TetTag cFos reporter mice, immunized with MOG_35–55_ peptide, showed robust GFP signals in the SC 5 d after removal of doxycycline (Fig. 1). The increase was detected only in symptomatic EAE mice (Fig. 1*c*), indicating that EAE insults produced c-Fos activation in the CNS. To understand the spatio-temporal c-Fos activation pattern, EAE SCs were processed by a tissue-clearing technique, iDISCO (Renier et al., 2014), combined with serial confocal images (Fig. 2*a–h*; Videos 1–7). Control SC from naive mice displayed sparse GFP signals even 5 d after doxycycline removal (Video 1). After 1 d post-onset (1 dpo), GFP^+^ nuclei appeared in the periphery of white matter tracts near associated blood vessels (Video 2). The GFP signals expanded along the rostral-caudal axis and penetrated into the white matter parenchyma over time, appearing most prominently near anatomically known major blood vessels including the anterior, posterior, and posterolateral spinal vessels (Fig. 2, Videos 3 and 4). Importantly, the number of GFP^+^ nuclei (Fig. 2*i*, *j*) and the average size of GFP^+^ signals (Fig. 2*k*, *l*) increased significantly over time, revealing that GFP^+^ nuclei aggregated to form clusters in EAE SCs at 5 dpo. These results identified robust c-Fos activation in cells near major CNS blood vessels in peripheral white matter tracts, followed by inward expansion centrally, into gray matter.

Video 1. 3D video of control spinal cord shown in Fig. 2a, b. 10.1523/ENEURO.0239-18.2018.video.1

Video 2. 3D video of EAE spinal cord at 1 dpo shown in Fig. 2c, d. 10.1523/ENEURO.0239-18.2018.video.2

Video 3. 3D video of EAE spinal cord at 3 dpo shown in Fig. 2e, f. 10.1523/ENEURO.0239-18.2018.video.3

Video 4. 3D video of EAE spinal cord at 5 dpo shown in Fig. 2g, h. 10.1523/ENEURO.0239-18.2018.video.4

Video 5. 3D video of WT^fos^ EAE spinal cord at 5 dpo shown in Fig. 4a, b. 10.1523/ENEURO.0239-18.2018.video.5

Video 6. 3D video of S1P_1_-AsCKO^fos^ EAE spinal cord at 5 dpo shown in Fig. 4c, d. 10.1523/ENEURO.0239-18.2018.video.6

Video 7. 3D video of FTY720-treated WT^fos^ EAE spinal cord at 5 dpo shown in Fig. 2e, f. 10.1523/ENEURO.0239-18.2018.video.7

### c-Fos^+^ cells are astrocytes: *ieAstrocytes*

**Figure 3.**
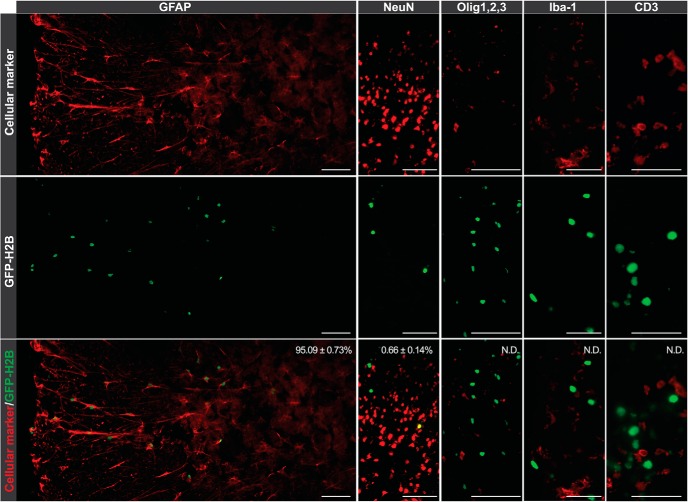
Immunolabeling identifies astrocytes as major c-Fos-activated cells in EAE SC. Immunolabeling for GFAP, NeuN, Olig1,2,3, Iba1, CD3, and GFP on SC sections, as indicated. Percentage overlap as indicated. N.D., not detected. *n* = 3 animals. Scale bar, 100 μm (GFAP, NeuN, and Olig1,2,3) and 50 μm (Iba-1 and CD3).

To determine the identities of GFP^+^ cell types, immunolabeling for various cellular markers was performed on 5-dpo SC sections. Strikingly, a vast majority of GFP^+^ nuclei (95.09 ± 0.73%, *n* = 3) were found to colabel with glial fibrillary acidic protein (GFAP; Fig. 3). By contrast, <1% of GFP^+^ nuclei (0.66% ± 0.14%, *n* = 3) colocalized with a neuronal nuclear marker (NeuN). Other assessed cell types included microglia, oligodendrocytes, and T lymphocytes, none of which showed colocalization with GFP signals (Fig. 3), whereas GFP^+^CD3^+^ T cells were found outside of the CNS within the peripheral lymph nodes of EAE mice, demonstrating their ability to report c-Fos (data not shown). The dominance of EAE-induced GFP^+^ astrocytes that reported transcription of the c-Fos IEG during disease development supported a functional classification for these astrocytes, which were therefore called immediate-early astrocytes (*ieAstrocytes*). At any given time following EAE challenge, *ieAstrocytes* could be distinguished from cells that did not show evidence of c-Fos activation, quiescent astrocytes (*qAstrocytes*).

10.1523/ENEURO.0239-18.2018.f3-1 Figure 3-1 Lower-magnification view of GFAP stained WT^fos^ EAE spinal cords. Immunolabeling identified astrocytes as the primary cell type in c-Fos–activated cells in EAE SC. Scale bar, 500 μm. Download Figure 3-1, JPG file.

### S1P_1_ inhibition reduces formation of EAE-induced *ieAstrocytes*

**Figure 4.**
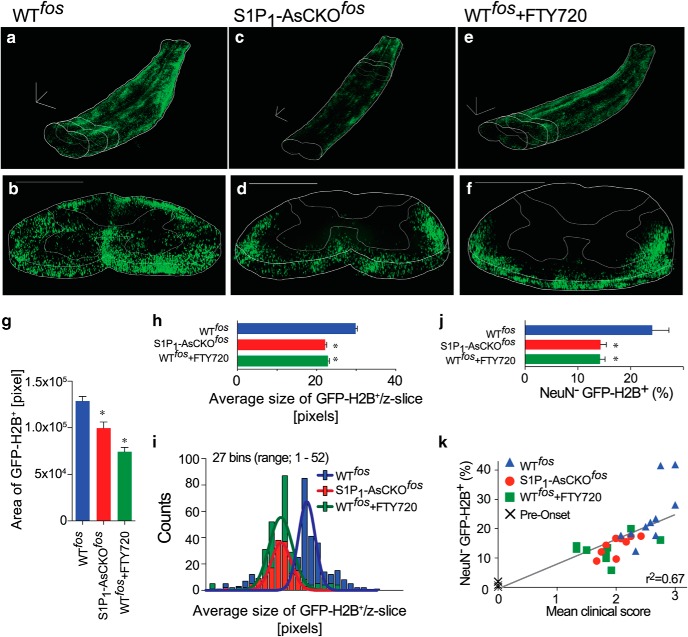
Genetic and pharmacological removal of S1P_1_ suppresses *ieAstrocyte* formation. ***a***–***f***, Representative 3D imaging of lower (lumbar region) SCs in EAE-induced WT^fos^ (***a*** and ***b***), S1P_1_-AsCKO^fos^ (***c*** and ***d***), and WT^fos^+FTY720 (***e*** and ***f***) mice of 5 dpo (dpo). ***a***, ***c***, ***e***, 3D-reconstructed images. ***b***, ***d***, ***f***, Coronal sections. Scale bar, 500 μm. ***g***, Area of GFP-H2B^+^ signals in the spinal cords (mean ± SEM, data were compiled from 303, 151, and 273 *z*-slices from *n* = 3, 2, and 3 animals, respectively, *p < 0.01 versus WT^fos^, Kruskal–Wallis test with Dunn’s *post hoc* test). ***h***, Average size of GFP-H2B^+^ signals (mean ± SEM, *p < 0.001 versus WT^fos^, Kruskal–Wallis test with Dunn’s *post hoc* test, data were compiled from 303, 151, and 273 *z*-slices from 3, 2, and 3 animals, respectively). ***i***, Distribution of the average size of GFP-H2B^+^ signals per *z*-slice shown in ***h***. Lines represent Gaussian fits to the data. ***j***, *ieAstrocyte* (NeuN^–^GFP^+^) populations in EAE SCs were determined by FCM (mean ± SEM, *n* = 10, 9, and 9 animals, *p < 0.05 versus WT^fos^ by one-way ANOVA with Bonferroni’s multiple comparisons test). ***k***, Correlation between *ieAstrocyte* (NeuN^–^GFP^+^) populations and clinical score. Each point represents a single animal.

To test whether S1P_1_ signaling influenced formation of *ieAstrocytes*, we crossed astrocyte-specific S1P_1_-KO mice (S1P_1_-AsCKO; S1P_1_^flox/flox^ mice harboring human GFAP promoter-driven Cre transgene; Choi et al., 2011) with the TetTag-c-Fos reporter mice to produce S1P_1_-AsCKO^fos^ (S1P_1_-AsCKO × TetTag/S1P_1_^flox/flox^) and WT^fos^ (TetTag/S1P_1_^flox/flox^) mice, which were then challenged with EAE. For the FTY720 treatment group, FTY720 (1.0 mg/kg) was orally administered from defined dpo (dpo = 0, clinical score ≥ 1.0). GFP^+^ signals at 5 dpo in S1P_1_-AsCKO^fos^ and FTY720-treated WT^fos^ (WT^fos^+FTY720) mice were attenuated compared with WT^fos^ controls (Fig. 4*a–f* and Videos 5–7). GFP^+^ signals were observed in gray matter of WT^fos^ controls (Fig. 4*b* and Video 5), while they were rarely found in S1P_1_-AsCKO^fos^ and WT^fos^+FTY720 mice (Fig. 4*d*, *f* and Videos 6 and 7). These were accompanied by a significant loss of *ieAstrocyte* numbers (Fig. 4 *g*) and the *ieAstrocyte* clusters (Fig. 4*h*, *i*) in S1P_1_-AsCKO^fos^ and WT^fos^+FTY720 mice compared with WT^fos^ controls. Flow cytometric (FCM) analysis of *ieAstrocytes* (DAPI-gated, NeuN^–^GFP^+^ populations) from the SCs of EAE mice revealed a significant decrease of *ieAstrocytes* (Fig. 4*j*) and a linear correlation of increasing *ieAstrocytes* with worsening clinical scores (Fig. 4*k*). These results link *ieAstrocyte* formation to EAE disease progression, both of which can be modified by S1P_1_ signaling.

## Discussion

The present study identified a functionally defined *in vivo* population of astrocytes, *ieAstrocytes*, as the earliest and predominant cell type responding to EAE insult. Surprisingly, other cell types were not functionally activated to a similar extent within the CNS. Moreover, *ieAstrocyte* formation increased with disease severity, and in part required S1P-S1P_1_ signaling.

IEGs as typified by c-Fos have been used as functional markers of cellular activation (Herrera and Robertson, 1996), particularly in the nervous system, where c-Fos was used as a transynaptic marker for neuronal activity (Bullitt, 1990), and most commonly relied on immunolabeling. In the present study, we used a TetTag c-Fos reporter system that is remarkable in tracking historical neural activity by labeling cells with stable GFP, compared with merely capturing momentary snapshots by classical c-Fos immunolabeling. This system historically traced the cells that experienced c-Fos expression during challenges, which identified a functional subset of neurons in learning and memory (Matsuo et al., 2008; Tayler et al., 2011) and functional astrocytes in EAE (Fig. 2). Although several studies identified c-Fos immunoreactivity in neurons with exclusion of c-Fos expression in astrocytes (Staiger et al., 2002; Wang et al., 2009; Lecrux et al., 2011), it was demonstrated in cultured astrocytes stimulated with excitatory amino acids (McNaughton and Hunt, 1992) and S1P (Yester et al., 2015), as well as in *in vivo* astrocytes, such as in electrical sensorimotor cortex stimulation (Morishita et al., 2011) and Alzheimer’s brains (Anderson et al., 1994). Moreover, results from direct activation of engineered Gαi-coupled GPCRs expressed in *in vivo* astrocytes using designer receptors exclusively activated by designer drugs (DREADDs; Chai et al., 2017), concur with our results that Gαi-coupled S1P_1_ signaling functionally activates c-Fos in astrocytes *in vivo* (Fig. 3).

Astrocytes are the most abundant cells in the CNS (Burda and Sofroniew, 2014; Farmer and Murai, 2017). They have been widely considered as bystanders in neuroinflammatory diseases (Rossi et al., 2007), responding secondarily under disease conditions to become reactive astrocytes that were originally defined by their hypertrophic morphology and increased GFAP expression, compared with quiescent *qAstrocytes* (Burda and Sofroniew, 2014; Hubbard and Binder, 2016). A recently proposed classification of A1 reactive astrocytes added transcriptomic signatures to the reactive astrocyte populations (Liddelow et al., 2017). By contrast, *ieAstrocytes* are defined (1) functionally (via c-Fos expression) and (2) temporally (in *vivo* during EAE development and progression) (Fig. 4). Considering the nature of immediate-early and transient c-Fos expression (Herrera and Robertson, 1996), *ieAstrocytes* appear to differ from reactive astrocytes, possibly representing an intermediate/transition state between *qAstrocytes* and reactive astrocytes, which moreover is partly driven by S1P signaling (Fig. 4). A possible scenario is that during EAE development, blood-borne S1P carried by albumin and apolipoprotein M (Blaho et al., 2015) at sufficient concentrations (∼µm) to activate S1P receptors (Tsai and Han, 2016) extravasates across a disrupted blood-brain barrier (Alvarez et al., 2011) to reach perivascular astrocytes in the spinal cord (Fig. 2 and Videos 1–7), resulting in direct activation of *qAstrocytes* to initiate *ieAstrocyte* formation. An alternative, but not mutually exclusive scenario, is the formation of A1 reactive astrocytes requiring microglial cytokine production (IL-1α, TNFα, and C1q) to activate astrocytes secondarily (Liddelow et al., 2017), to give rise to both *ieAstrocytes* and reactive astrocytes. The determination of the involved activation pathways will require further study.

Therapeutic reduction of S1P_1_ signaling is thought to reduce pathogenic lymphocyte trafficking into the brain, which has been proposed as the immunologic MOA of fingolimod (Mandala et al., 2002; Chun and Hartung, 2010; Arnon et al., 2011; Cohen and Chun, 2011; Chun et al., 2018). However, a direct CNS MOA involving astrocytes (Choi et al., 2011; Groves et al., 2013) receives further support through the dominant presence of *ieAstrocytes,* whose increasing numbers correlated with disease severity (Fig. 4*k*), and from the fact that genetic removal or pharmacological inhibition by fingolimod of S1P_1_ signaling (Choi et al., 2011; Groves et al., 2013) reduced both *ieAstrocytes* and disease severity. Notably, complete S1P_1_ removal did not eliminate *ieAstrocytes*, indicating that other factors must be involved in their activation. In this light, other MS drugs that have been proposed to access a direct CNS MOA distinct from S1P receptor modulation in EAE, including interferon β (Rothhammer et al., 2016) and dimethyl fumarate (Al-Jaderi and Maghazachi, 2016), might also reduce *ieAstrocyte* formation. Although these relationships remain to be determined, they theoretically support combination therapies that could more completely prevent *ieAstrocyte* formation. A next-generation S1P receptor modulator, siponimod (BAF312), that also engages S1P_1_ (and S1P_5_), showed positive results in a phase III secondary progressive MS trial (Kappos et al., 2018), which is consistent with a CNS MOA involving *ieAstrocytes*, while other related agents like ozanimod (Cohen et al., 2016), etrasimod (Buzard et al., 2014), and ponesimod (Bolli et al., 2010; Vaclavkova et al., 2014)—having similar, albeit distinct, S1P receptor engagement properties—may also have direct CNS effects in MS through *ieAstrocytes*. Overall, our results support a previously unrecognized pathogenic role of functionally defined astrocytes *in vivo*, which could reveal new disease mechanisms and therapeutic targets accessed through *ieAstrocytes.*

## Synthesis

Reviewing Editor: Alfonso Araque, University of Minnesota Decisions are customarily a result of the Reviewing Editor and the peer reviewers coming together and discussing their recommendations until a consensus is reached. When revisions are invited, a fact-based synthesis statement explaining their decision and outlining what is needed to prepare a revision will be listed below. The following reviewer(s) agreed to reveal their identity: NONE.

While both reviewers have found that the manuscript presents novel and interesting results that will be will inform future efforts for the treatment of neuroinflammatory diseases. They, however, expressed some minor concerns. I concur with their assessments and I also believe that addressing these minor issues, which seems to be feasibly done in a reasonable short time, will enhance the strength of the manuscript.

I am looking forward to receiving a revised version of the manuscript.

Sincerely

Specific comments of the reviewers:

Reviewer 1.

General comment:

This manuscript identifies a population of astrocytes that express c-Fos upon the induction of inflammation (in the context of EAE), and shows that c-Fos activation is reduced upon loss of S1P1 signaling. The results will inform efforts to treat neuroinflammatory disease using a panoply of drugs that modulate S1P signaling.

Specific comments:

This manuscript describes c-Fos activation in a subpopulation of astrocytes upon inflammation (EAE), and demonstrates that c-Fos activation is regulated by S1P1 signaling in astrocytes. The results will inform efforts to treat neuroinflammatory disease using a panoply of drugs that modulate S1P signaling.

Minor concerns:

1. Fig. 1c: Please define n=4 -- 4 mice or 4 sections (or is this done by flow cytometry)? In all cases, compilations should include more than one mouse.

1. Fig. 2i: The compilation should show more than one mouse for each timepoint.

2. Fig. 2j: How is ‘Cluster formation (%)’ defined? Also what data are compiled in the bar graph?

3. Fig 3: What data are compiled to calculate the mean/SEM?

4. Fig. 4g-i: What is being compiled?

Reviewer 2.

General comment:

The study advances our knowledge on the signaling cascades associated with the EAE model of MS. The authors highlight the early activation of fos primarily in astrocytes. Using an advanced genetic tool, they functionally characterize a subpopulation of reactive astrocytes in the spinal cord, which could be a target for future research.

Specific comments:

The manuscript “A functionally defined in vivo astrocyte population identified by c-Fos activation in a mouse model of multiple sclerosis modulated by S1P signaling: immediate-early astrocytes (ieAstrocytes)” describes an interesting study. Using an advanced genetic tool, the authors convincingly show that EAE-mediated activation of c-fos in the spinal cord primarily occurs in astrocytes (95 %). In addition, the authors provide additional data suggesting that S1P receptor signaling modulates the astroglia activation.

In general, the study is carefully performed and well written. The results will be helpful to the community working on MS.

There are only minor comments:

In all figures, the use of blue or green colors on a black background is contra-productive, Well, DAPI is regarded as blue and GFP as green, but the signal is detected by an opto-electronic sensor. The color afterwards depends on the color look-up table. Please use grey and for the single channels and the data will be much easier recognized by the reader. Also the movies should be in presented with a grey scale LUT.

The results largely depend on figure 3, upper left image. Please provide complete overviews of the spinal cord in which also white and grey matter areas can be distinguished.

## References

[B1] Acosta C, Anderson HD, Anderson CM (2017) Astrocyte dysfunction in Alzheimer disease. J Neurosci Res 95:2430–2447. 10.1002/jnr.24075 28467650

[B2] Al-Jaderi Z, Maghazachi AA (2016) Utilization of dimethyl fumarate and related molecules for treatment of multiple sclerosis, cancer, and other diseases. Front Immunol 7:278 10.3389/fimmu.2016.00278 27499754 PMC4956641

[B3] Alvarez JI, Cayrol R, Prat A (2011) Disruption of central nervous system barriers in multiple sclerosis. Biochim Biophys Acta 1812:252–264. 10.1016/j.bbadis.2010.06.017 20619340

[B4] Anderson AJ, Cummings BJ, Cotman CW (1994) Increased immunoreactivity for Jun- and Fos-related proteins in Alzheimer’s disease: association with pathology. Exp Neurol 125:286–295. 10.1006/exnr.1994.1031 8313943

[B5] Arnon TI, Xu Y, Lo C, Pham T, An J, Coughlin S, Dorn GW, Cyster JG (2011) GRK2-dependent S1PR1 desensitization is required for lymphocytes to overcome their attraction to blood. Science 333:1898–1903. 10.1126/science.1208248 21960637 PMC3267326

[B6] Blaho VA, Galvani S, Engelbrecht E, Liu C, Swendeman SL, Kono M, Proia RL, Steinman L, Han MH, Hla T (2015) HDL-bound sphingosine-1-phosphate restrains lymphopoiesis and neuroinflammation. Nature 523:342–346. 10.1038/nature14462 26053123 PMC4506268

[B7] Bolli MH, Abele S, Binkert C, Bravo R, Buchmann S, Bur D, Gatfield J, Hess P, Kohl C, Mangold C, et al . (2010) 2-Imino-thiazolidin-4-one derivatives as potent, orally active S1P1 receptor agonists. J Med Chem 53:4198–4211. 10.1021/jm100181s 20446681

[B8] Bullitt E (1990) Expression of C-fos-like protein as a marker for neuronal activity following noxious stimulation in the rat. J Comp Neur 296:517–530. 10.1002/cne.902960402 2113539

[B9] Burda JE, Sofroniew MV (2014) Reactive gliosis and the multicellular response to CNS damage and disease. Neuron 81:229–248. 10.1016/j.neuron.2013.12.034 24462092 PMC3984950

[B10] Buzard DJ, Kim SH, Lopez L, Kawasaki A, Zhu X, Moody J, Thoresen L, Calderon I, Ullman B, Han S, et al (2014) Discovery of APD334: design of a clinical stage functional antagonist of the sphingosine-1-phosphate-1 receptor. ACS Med Chem Lett 5:1313–1317. 10.1021/ml500389m 25516790 PMC4265817

[B11] Cekanaviciute E, Buckwalter MS (2016) Astrocytes: integrative regulators of neuroinflammation in stroke and other neurological diseases. Neurotherapeutics 13:685–701. 10.1007/s13311-016-0477-8 27677607 PMC5081110

[B12] Chai H, Diaz-Castro B, Shigetomi E, Monte E, Octeau JC, Yu X, Cohn W, Rajendran PS, Vondriska TM, Whitelegge JP, et al (2017) Neural circuit-specialized astrocytes: transcriptomic, proteomic, morphological, and functional evidence. Neuron 95:531–549.e539. 10.1016/j.neuron.2017.06.029 28712653 PMC5811312

[B13] Choi JW, Gardell SE, Herr DR, Rivera R, Lee CW, Noguchi K, Teo ST, Yung YC, Lu M, Kennedy G, et al (2011) FTY720 (fingolimod) efficacy in an animal model of multiple sclerosis requires astrocyte sphingosine 1-phosphate receptor 1 (S1P1) modulation. Proc Natl Acad Sci USA 108:751–756. 10.1073/pnas.1014154108 21177428 PMC3021041

[B15] Chun J, Hartung HP (2010) Mechanism of action of oral fingolimod (FTY720) in multiple sclerosis. Clin Neuropharmacol 33:91–101. 10.1097/WNF.0b013e3181cbf825 20061941 PMC2859693

[B14] Chun J, Brinkmann V (2011) A mechanistically novel, first oral therapy for multiple sclerosis: the development of fingolimod (FTY720, Gilenya). Discov Med 12:213–228. 21955849 PMC3694567

[B16] Chun J, Kihara Y, Jonnalagadda D, Blaho VA (2018) Fingolimod: lessons learned and new opportunities for treating multiple sclerosis and other disorders. Ann Rev Pharmacol Toxicol, in press. 10.1146/annurev-pharmtox-010818-021358 PMC6392001 30625282

[B18] Cohen JA, Chun J (2011) Mechanisms of fingolimod’s efficacy and adverse effects in multiple sclerosis. Ann Neurol 69:759–777. 10.1002/ana.22426 21520239

[B17] Cohen JA, Arnold DL, Comi G, Bar-Or A, Gujrathi S, Hartung JP, Cravets M, Olson A, Frohna PA, Selmaj KW, et al (2016) Safety and efficacy of the selective sphingosine 1-phosphate receptor modulator ozanimod in relapsing multiple sclerosis (RADIANCE): a randomised, placebo-controlled, phase 2 trial. Lancet Neurol 15:373–381. 10.1016/S1474-4422(16)00018-1 26879276

[B19] Colombo E, Di Dario M, Capitolo E, Chaabane L, Newcombe J, Martino G, Farina C (2014) Fingolimod may support neuroprotection via blockade of astrocyte nitric oxide. Ann Neurol 76:325–337. 10.1002/ana.24217 25043204

[B20] Dusaban SS, Chun J, Rosen H, Purcell NH, Brown JH (2017) Sphingosine 1-phosphate receptor 3 and RhoA signaling mediate inflammatory gene expression in astrocytes. J Neuroinflammation 14:111 10.1186/s12974-017-0882-x 28577576 PMC5455202

[B21] Eun SY, Hong YH, Kim EH, Jeon H, Suh YH, Lee JE, Jo C, Jo SA, Kim J (2004) Glutamate receptor-mediated regulation of c-fos expression in cultured microglia. Biochem Biophys Res Commun 325:320–327. 10.1016/j.bbrc.2004.10.035 15522236

[B22] Farmer WT, Murai K (2017) Resolving astrocyte heterogeneity in the CNS. Front Cell Neurosci 11:300. 10.3389/fncel.2017.00300 29021743 PMC5623685

[B23] Gardell SE, Dubin AE, Chun J (2006) Emerging medicinal roles for lysophospholipid signaling. Trends Mol Med 12:65–75. 10.1016/j.molmed.2005.12.001 16406843

[B24] González H, Pacheco R (2014) T-cell-mediated regulation of neuroinflammation involved in neurodegenerative diseases. J Neuroinflammation 11:201 10.1186/s12974-014-0201-8 25441979 PMC4258012

[B25] Graham JM (2001). Isolation of nuclei and nuclear membranes from animal tissues In Current Protocols in Cell Biology. Hoboken, NJ: Wiley. 10.1002/0471143030.cb0310s12 18228353

[B26] Groves A, Kihara Y, Chun J (2013) Fingolimod: direct CNS effects of sphingosine 1-phosphate (S1P) receptor modulation and implications in multiple sclerosis therapy. J Neurol Sci 328:9–18. 10.1016/j.jns.2013.02.011 23518370 PMC3640626

[B27] Herr DR, Chun J (2007) Effects of LPA and S1P on the nervous system and implications for their involvement in disease. Curr Drug Targets 8:155–167. 17266539 10.2174/138945007779315669

[B28] Herrera DG, Robertson HA (1996) Activation of c-fos in the brain. Prog Neurobiol 50:83–107. 8971979 10.1016/s0301-0082(96)00021-4

[B29] Hubbard AJ, Binder DK (2016). Chapter 1. History of astrocytes In Astrocytes and Epilepsy. Cambridge, MA: Academic Press, pp. 1–38.

[B30] Kappos L, Bar-Or A, Cree BAC, Fox RJ, Giovannoni G, Gold R, Vermersch P, Arnold DL, Arnould S, Scherz T, et al (2018) Siponimod versus placebo in secondary progressive multiple sclerosis (EXPAND): a double-blind, randomised, phase 3 study. Lancet 391:1263–1273. 10.1016/S0140-6736(18)30475-6 29576505

[B32] Kihara Y, Matsushita T, Kita Y, Uematsu S, Akira S, Kira J-i, Ishii S, Shimizu T (2009) Targeted lipidomics reveals mPGES-1-PGE2 as a therapeutic target for multiple sclerosis. Proc Natl Acad Sci USA 106:21807–21812. 10.1073/pnas.0906891106 19995978 PMC2789753

[B31] Kihara Y, Maceyka M, Spiegel S, Chun J (2014) Lysophospholipid receptor nomenclature review: IUPHAR Review 8. Br J Pharmacol 171:3575–3594. 10.1111/bph.12678 24602016 PMC4128058

[B33] Kihara Y, Mizuno H, Chun J (2015) Lysophospholipid receptors in drug discovery. Exp Cell Res 333:171–177. 10.1016/j.yexcr.2014.11.020 25499971 PMC4408218

[B34] Lau LF, Nathans D (1987) Expression of a set of growth-related immediate early genes in BALB/c 3T3 cells: coordinate regulation with c-fos or c-myc. Proc Natl Acad Sci USA 84:1182–1186. 3469660 10.1073/pnas.84.5.1182 PMC304390

[B35] Lecrux C, Toussay X, Kocharyan A, Fernandes P, Neupane S, Levesque M, Plaisier F, Shmuel A, Cauli B, Hamel E (2011) Pyramidal neurons are “neurogenic hubs” in the neurovascular coupling response to whisker stimulation. J Neurosci 31:9836–9847. 10.1523/JNEUROSCI.4943-10.2011 21734275 PMC6703330

[B36] Lee CW, Choi JW, Chun J (2010) Neurological S1P signaling as an emerging mechanism of action of oral FTY720 (fingolimod) in multiple sclerosis. Arch Pharm Res 33:1567–1574. 10.1007/s12272-010-1008-5 21052934

[B37] Liddelow SA, Guttenplan KA, Clarke LE, Bennett FC, Bohlen CJ, Schirmer L, Bennett ML, Münch AE, Chung WS, Peterson TC, et al (2017) Neurotoxic reactive astrocytes are induced by activated microglia. Nature 541:481–487. 10.1038/nature21029 28099414 PMC5404890

[B38] Mandala S, Hajdu R, Bergstrom J, Quackenbush E, Xie J, Milligan J, Thornton R, Shei GJ, Card D, Keohane C, et al (2002) Alteration of lymphocyte trafficking by sphingosine-1-phosphate receptor agonists. Science 296:346–349. 10.1126/science.1070238 11923495

[B39] Matsuo N, Reijmers L, Mayford M (2008) Spine-type-specific recruitment of newly synthesized AMPA receptors with learning. Science 319:1104–1107. 10.1126/science.1149967 18292343 PMC2692967

[B40] McNaughton LA, Hunt SP (1992) Regulation of gene expression in astrocytes by excitatory amino acids. Brain Res Mol Brain Res 16:261–266. 1337935 10.1016/0169-328x(92)90234-3

[B41] Moon E, Han JE, Jeon S, Ryu JH, Choi JW, Chun J (2015) Exogenous S1P exposure potentiates ischemic stroke damage that is reduced possibly by inhibiting S1P receptor signaling. Mediators Inflamm 2015:492659 10.1155/2015/492659 26576074 PMC4630407

[B42] Morishita T, Yamashita A, Katayama Y, Oshima H, Nishizaki Y, Shijo K, Fukaya C, Yamamoto T (2011) Chronological changes in astrocytes induced by chronic electrical sensorimotor cortex stimulation in rats. Neurol Med Chir (Tokyo) 51:496–502. 10.2176/nmc.51.496 21785243

[B43] Muir DA, Compston DA (1996) Growth factor stimulation triggers apoptotic cell death in mature oligodendrocytes. J Neurosci Res 44:1–11. 10.1002/(SICI)1097-4547(19960401)44:1<1::AID-JNR1>3.0.CO;2-L 8926624

[B44] Mullershausen F, Craveiro LM, Shin Y, Cortes-Cros M, Bassilana F, Osinde M, Wishart WL, Guerini D, Thallmair M, Schwab ME, Sivasankaran R, Seuwen K, Dev KK (2007) Phosphorylated FTY720 promotes astrocyte migration through sphingosine-1-phosphate receptors. J Neurochem 102:1151–1161. 10.1111/j.1471-4159.2007.04629.x 17488279

[B45] Noguchi K, Chun J (2011) Roles for lysophospholipid S1P receptors in multiple sclerosis. Crit Rev Biochem Mol Biol 46:2–10. 10.3109/10409238.2010.522975 20979571

[B46] Noseworthy JH, Lucchinetti C, Rodriguez M, Weinshenker BG (2000) Multiple sclerosis. N Engl J Med 343:938–952. 10.1056/NEJM200009283431307 11006371

[B47] Proia RL, Hla T (2015) Emerging biology of sphingosine-1-phosphate: its role in pathogenesis and therapy. J Clin Invest 125:1379–1387. 10.1172/JCI76369 25831442 PMC4409021

[B48] Renier N, Wu Z, Simon DJ, Yang J, Ariel P, Tessier-Lavigne M (2014) iDISCO: a simple, rapid method to immunolabel large tissue samples for volume imaging. Cell 159:896–910. 10.1016/j.cell.2014.10.010 25417164

[B49] Rossi DJ, Brady JD, Mohr C (2007) Astrocyte metabolism and signaling during brain ischemia. Nat Neurosci 10:1377–1386. 10.1038/nn2004 17965658 PMC8906499

[B50] Rothhammer V, Mascanfroni ID, Bunse L, Takenaka MC, Kenison JE, Mayo L, Chao CC, Patel B, Yan R, Blain M, et al (2016) Type I interferons and microbial metabolites of tryptophan modulate astrocyte activity and central nervous system inflammation via the aryl hydrocarbon receptor. Nat Med 22:586–597. 10.1038/nm.4106 27158906 PMC4899206

[B51] Rouach N, Pébay A, Même W, Cordier J, Ezan P, Etienne E, Giaume C, Tencé M (2006) S1P inhibits gap junctions in astrocytes: involvement of G and Rho GTPase/ROCK. Eur J Neurosci 23:1453–1464. 10.1111/j.1460-9568.2006.04671.x 16553609

[B52] Staiger JF, Masanneck C, Bisler S, Schleicher A, Zuschratter W, Zilles K (2002) Excitatory and inhibitory neurons express c-Fos in barrel-related columns after exploration of a novel environment. Neuroscience 109:687–699. 10.1016/S0306-4522(01)00501-2 11927151

[B53] Tayler K, Lowry E, Tanaka K, Levy B, Reijmers L, Mayford M, Wiltgen B (2011) Characterization of NMDAR-independent learning in the hippocampus. Front Behav Neurosci 5:10.3389/fnbeh.2011.00028 PMC3099364 21629769

[B54] Trépanier MO, Hopperton KE, Mizrahi R, Mechawar N, Bazinet RP (2016) Postmortem evidence of cerebral inflammation in schizophrenia: a systematic review. Mol Psychiatry 21:1009–1026. 10.1038/mp.2016.90 27271499 PMC4960446

[B55] Tsai HC, Han MH (2016) Sphingosine-1-phosphate (S1P) and S1P signaling pathway: therapeutic targets in autoimmunity and inflammation. Drugs 76:1067–1079. 10.1007/s40265-016-0603-2 27318702

[B56] Vaclavkova A, Chimenti S, Arenberger P, Holló P, Sator PG, Burcklen M, Stefani M, D’Ambrosio D (2014) Oral ponesimod in patients with chronic plaque psoriasis: a randomised, double-blind, placebo-controlled phase 2 trial. Lancet 384:2036–2045. 10.1016/S0140-6736(14)60803-5 25127208

[B57] Wang W, Wang W, Mei X, Huang J, Wei Y, Wang Y, Wu S, Li Y (2009) Crosstalk between spinal astrocytes and neurons in nerve injury-induced neuropathic pain. PLoS One 4:e6973 10.1371/journal.pone.0006973 19759893 PMC2736402

[B58] Yester JW, Bryan L, Waters MR, Mierzenski B, Biswas DD, Gupta AS, Bhardwaj R, Surace MJ, Eltit JM, Milstien S, et al (2015) Sphingosine-1-phosphate inhibits IL-1-induced expression of C-C motif ligand 5 via c-Fos-dependent suppression of IFN-beta amplification loop. FASEB J 29:4853–4865. 10.1096/fj.15-275180 26246404 PMC4653053

