# Supplementary figures and images for: A Functionally Defined In Vivo Astrocyte Population Identified by c-Fos Activation in a Mouse Model of Multiple Sclerosis Modulated by S1P Signaling: Immediate-Early Astrocytes (ieAstrocytes)
Source: eNeuro. 2018 Sep 24;5(5):ENEURO.0239-18.2018. doi: 10.1523/ENEURO.0239-18.2018 (PMC6153337; doi:10.1523/ENEURO.0239-18.2018)

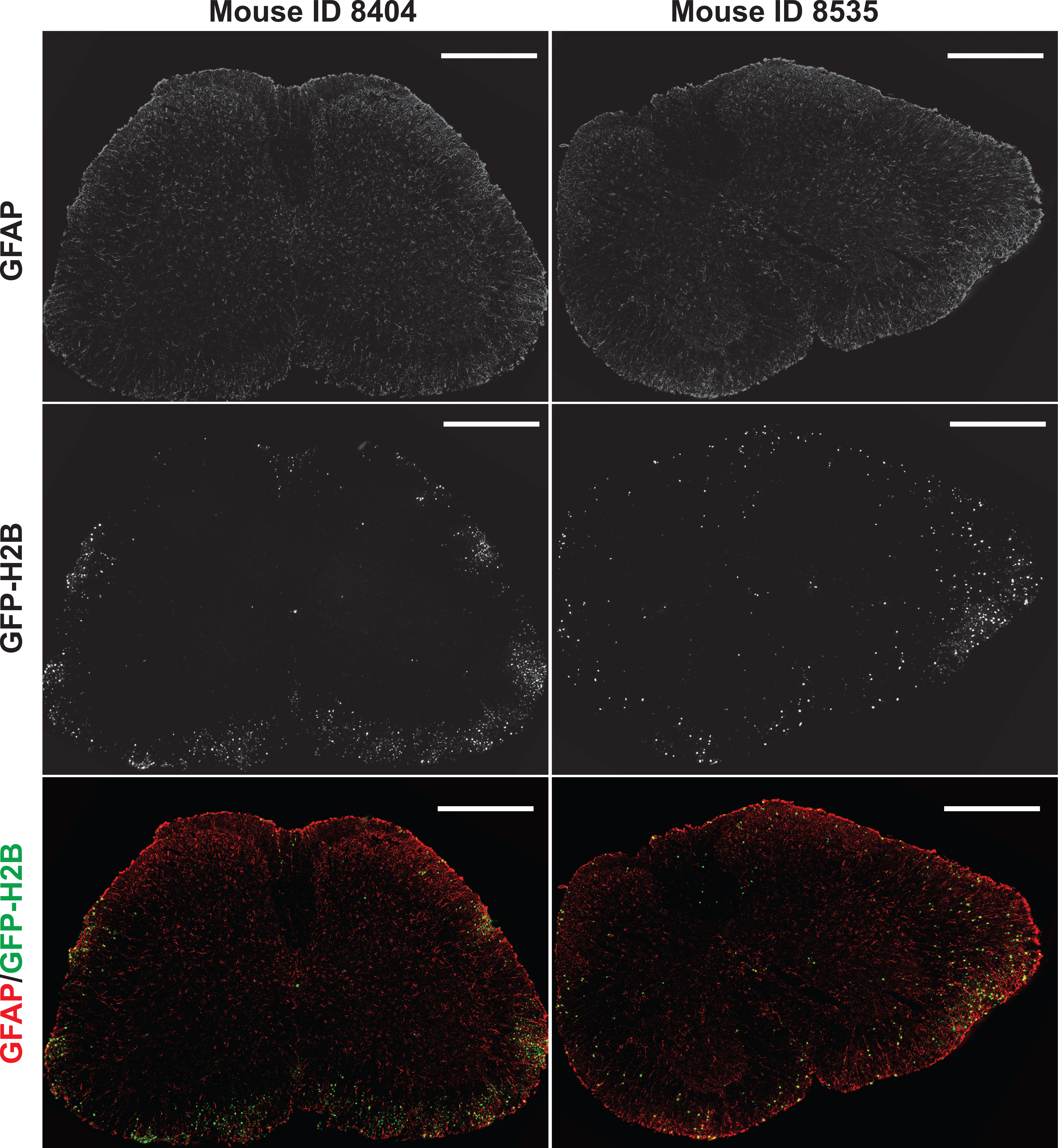

Supplement: Figure 3-1 — Lower-magnification view of GFAP stained WTfos EAE spinal cords. Immunolabeling identified astrocytes as the primary cell type in c-Fos–activated cells in EAE SC. Scale bar, 500 μm. Download Figure 3-1, JPG file. [file sup_enu-eN-TNC-0239-18-s01.jpg]
